# A Rare Recurrence of a Thyroglossal Duct Cyst Accompanied by Several Postoperative Complications in an Eight-Year-Old Boy: A Case Report

**DOI:** 10.7759/cureus.54870

**Published:** 2024-02-25

**Authors:** Ethan Dimock, Jithin John, Jatin Ahluwalia, Alise Haddad, Michael Haupert

**Affiliations:** 1 Otolaryngology, Oakland University William Beaumont School of Medicine, Auburn Hills, USA; 2 Otolaryngology, Detroit Medical Center, Detroit, USA; 3 Otolaryngology, Ascension Providence Hospital, Detroit, USA; 4 Otolaryngology, Corewell Health William Beaumont University Hospital, Royal Oak, USA

**Keywords:** pediatric head and neck surgery, thyroglossal duct cyst recurrence, sclerotherapy, otolaryngology, sistrunk procedure

## Abstract

A thyroglossal duct cyst (TGDC) is a fluid-filled mass in the neck resulting from the persistence of a duct from fetal development that typically regresses spontaneously. When it persists, it is most often removed in a surgical procedure known as a Sistrunk operation. This case study presents the intriguing case of an eight-year-old boy who presented to an otolaryngology clinic with both a recurrence of his TGDC, as well as several postoperative complications, after the Sistrunk operation was performed. After the initial procedure resulted in an incomplete removal of the TGDC, the patient was referred to Interventional Radiology for sclerotherapy. After several rounds of this treatment technique the cyst remnants still persisted along with their associated symptoms. Due to the very low likelihood of a recurrence being observed after surgical removal with subsequent sclerotherapy, the reappearance of the cyst raised several clinical questions. This report underscores the significance of a thorough evaluation and consideration of unique presentations when confronted with recurrent TGDCs.

## Introduction

The Sistrunk operation is a widely accepted surgical intervention within the field of otolaryngology due to its comprehensive approach in removing thyroglossal duct cysts (TGDCs) by not only removing the cyst, but also the middle part of the hyoid bone and the surrounding tissue. This is especially true when comparing its recurrence rate of 5.3% to a simple excision, which has a recurrence rate of 55.6% [1]. The most noteworthy difference between these two procedures is the fact that the Sistrunk operation involves the removal of the body of the hyoid bone to ensure that the full cyst is excised from the body. This is in contrast to a simple excision, where recurrence was common due to TGDC remnants that would often remain anchored to the hyoid bone [1]. The aforementioned statements all contributed to the procedure developed by Dr. George Sistrunk in the mid-1900s to eventually become the standard of care for this pathology within the field of otolaryngology [2]. Due to the typically high rate of success attributed to this procedure, physicians are especially concerned with the exceedingly rare occurrence of persistence of postoperative complications. This case examines the emerging technique of sclerotherapy in the treatment of recurrent TGDCs. Multiple attempts were made using this approach, but due to the persistence of the target tissue the more traditional approach of an “extended” Sistrunk procedure was ultimately performed. 

## Case presentation

This case delineates the clinical course of an eight-year-old Caucasian male patient presenting with a recurrent TGDC to an otolaryngology clinic in a hospital outside of Detroit, Michigan, United States. 

The initial manifestation of the TGDC occurred two years prior, characterized by a non-painful midline mass at the hyoid bone level, exacerbated with tongue protrusion, prompting suspicion of TGDC. Aside from these findings, the rest of the physical exam and review of systems was normal as he was noted to have speech appropriate for his age, a good quality of sleep, a good appetite, normal vitals, and a height and weight that fall within the normal range. This patient also did not have any prior significant past medical or surgical history. 

**Figure 1:**
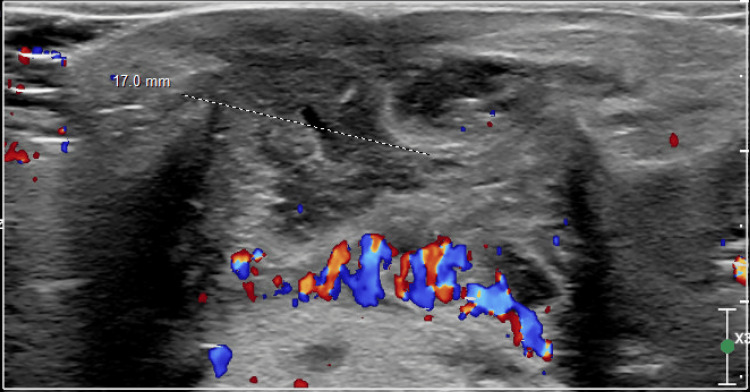
Ultrasound Demonstrating the Presence of a Thyroglossal Duct Cyst (TGDC)

The midline mass prompted a subsequent ultrasound which confirmed the diagnosis (Figure 1), leading to the implementation of a Sistrunk procedure for definitive intervention, involving the excision of the hyoid bone. The wound was closed with Steri-Strips and no drainage or redness was observed. The excised mass was sent to pathology for examination and the results were consistent with normal findings for an excised TGDC. The report described the mass as “fibromuscular tissue with patchy areas showing granulation tissue with mixed acute and chronic inflammatory cells associated with degenerated skeletal muscle fibers”. His mother was instructed to give the patient 9.3 mL of ibuprofen by mouth every six hours as needed for postoperative pain management, along with 5 mL of clindamycin by mouth every eight hours for 10 days for bacterial prophylaxis.

**Figure 2:**
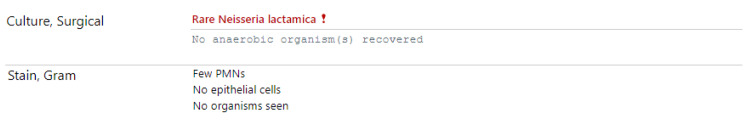
Surgical Culture Results PMN: Polymorphonuclear neutrophil

Six days after the procedure, the patient was reported to have “significant submental swelling” that was out of proportion to what is normally observed at this stage of the healing process. This prompted the ENT to supplement the drug regimen with sulfamethoxazole-trimethoprim at a dose of 10 mL by mouth every 12 hours for seven days. Three days after updating the drug regimen, he presented to the otolaryngology clinic for a follow-up visit displaying a "greenish" drainage at the incision site despite adherence to the sulfamethoxazole-trimethoprim antibiotics, necessitating a Penrose drain placement. A soft tissue biopsy showed the presence of a rare Neisseria lactamica (Figure 2) and the final diagnosis was a submental abscess. The abscess was drained, new dressings were placed, and the antibiotic regimen was continued.

**Figure 3:**
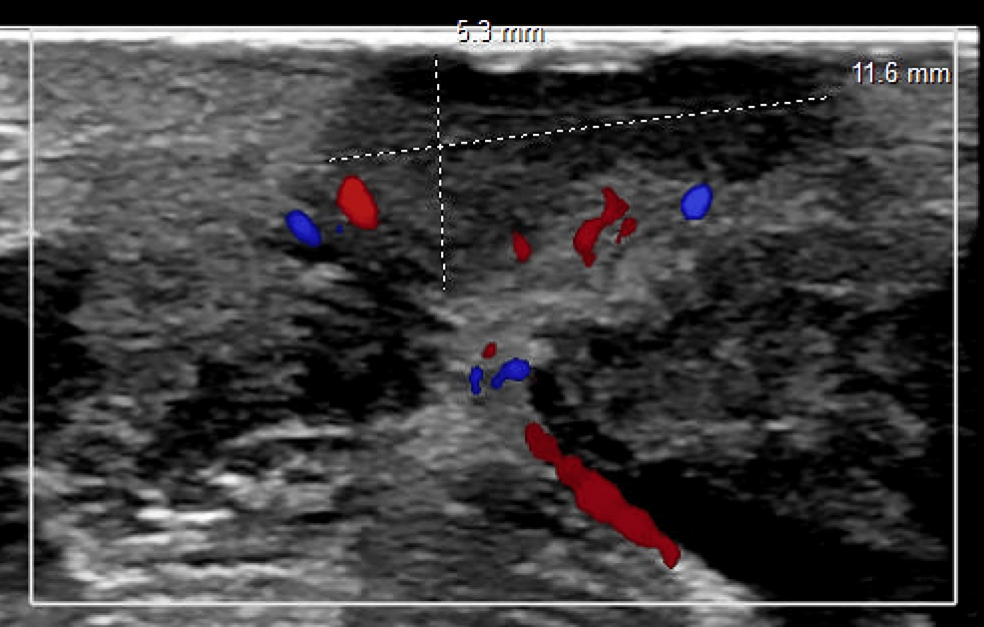
Ultrasound Demonstrating Fluid Collection at Site of Surgery

Persistent drainage and granulation tissue several weeks later prompted an ultrasound of the neck, which showed 2.5 cm of fluid that had collected in the area of the previous TGDC resection (Figure 3). The ENT then referred the patient to pediatric Interventional Radiology, where intravenous sclerotherapy with 100 mg doxycycline suspended in 8 mL sterile water with 1 mL contrast was initiated to shrink the residual TGDC that was determined to be present due to the persistent collection of fluid. This procedure occurred three months after the excision of the TGDC. A subsequent round of sclerotherapy was indicated four months after the first round due to persistent swelling of the neck with intermittent cyst rupture.

**Figure 4:**
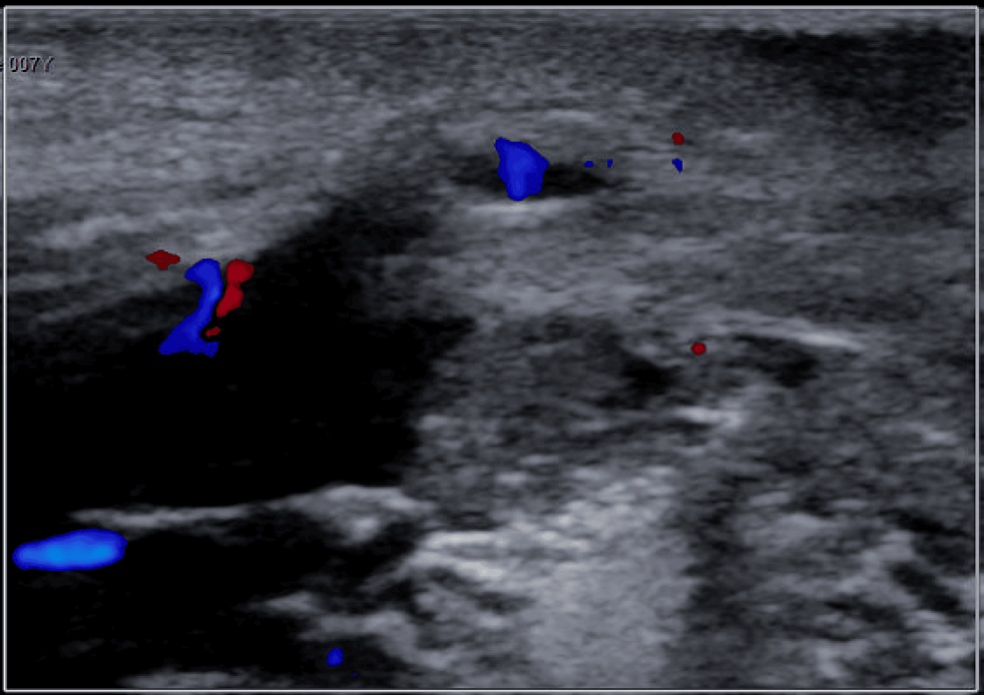
Ultrasound Demonstrating Areas of Thyroglossal Duct Cyst (TGDC) Persistence

Despite these interventions, symptoms endured, accompanied by mild weight loss over the next eight months. Ultrasound confirmation of TGDC recurrence (Figure 4) led to a second surgical removal procedure, performed successfully two months later. Notably, postoperative complications manifested as an apparent allergic reaction, managed with Benadryl (7 mg intravenous injection), steroids (4 mg intravenous injection), and subsequent antibiotic therapy with cefdinir (250 mg/5 ml by mouth).

Two weeks after the second TGDC excision surgery, the patient has a slight accumulation of fluid at the surgical site but is otherwise noted as healthy with a normal physical examination.

## Discussion

Thyroglossal duct cysts are often initially discovered as a visible midline mass on the anterior neck below the level of the hyoid bone [3]. This presentation is distinguished from other cervical masses, such as a branchial cleft cyst, due to the fact that it moves with both tongue protrusion and swallowing [3]. They occur in approximately 7% of the population worldwide and are distributed evenly between males and females [4]. They appear as a result of the failure of the closure of the embryological thyroglossal duct, which is an outgrowth of the primitive pharynx that develops during the third week of gestation [3]. The nonobservance of this involution is what leads to the subsequent clinical findings that are associated with a TGDC.

The Sistrunk procedure, developed by Dr. George Sistrunk, gained prominence in the medical community because it provided a robust solution to thyroglossal duct cysts by comprehensively addressing the issue, thus reducing the chances of reoccurrence [2]. Aided by the success of this mid-20th-century invention, this groundbreaking contribution has become the standard of care and has been widely accepted among healthcare professionals [2].

Due to the low rates of recurrence following the Sistrunk procedure, the circumstances of these reappearances are always carefully and holistically scrutinized. The most common cause for recurrence is an incomplete removal of the thyroglossal tract and its branching ductules during the primary procedure [3]. These ductules branch from the main duct and are difficult to detect during the operation [3]. Even a small amount of remaining tissue can lead to recurrence for several reasons. This includes the ability of the remaining tissue to grow and expand, the transformation of either pre-existing or dormant cysts, or the development of de novo cysts [3].

The most common method for treating all of the various forms of TGDC recurrence is an “extended” Sistrunk procedure. In a study by Pastore and Bartoli, this procedure was demonstrated to have a 100% success rate in eliminating postoperative complications and recurrences [5]. This study goes on to mention that this follow-up operation is both “highly effective and safe”. An alternate approach to treating recurrences, and the one that was initially attempted in this case study, is the emerging technique of sclerotherapy. This technique involves an interventional radiologist injecting a sclerosing agent into the cyst to induce sclerosis and resolve the cyst. It is less invasive than another procedure and involves a shorter postoperative recovery time.

Existing literature advises against the use of sclerotherapy as the primary method of treatment for pediatric TGDCs [6]. Kim and Chung describe cases where this was attempted, and ultimately failed, in a literature review that looked into the success rates of this approach [6]. However, current literature generally supports the use of sclerotherapy when treating the recurrence of the TGDC [7]. A case where this was attempted successfully in a pediatric patient is outlined by Ibrahim and Daniel, where they treat the recurrent cyst with doxycycline as the sclerosing agent [7].

Our case is unique because the approach that was taken is fully backed by existing literature, but the TGDC took years to ultimately resolve and required multiple modalities of treatment. To the best of our knowledge, there are no existing cases where each of these lines of action were subsequently attempted without a successful resolution of the cyst. It was not until the final follow-up procedure that the symptoms finally ceased. The patient was not only resistant to the initial interventional treatments, but also required multiple changes in antibiotic regimens and was found to have an infection with a rare strain of Neisseria lactamica at the site of the wound.

## Conclusions

Based on a thorough review of existing literature, this is the first case of a recurrence of a TGDC that persisted beyond all of the initial interventional methods. Typically the cyst and its associated symptoms are resolved with the initial Sistrunk procedure. This procedure has a small chance of failing and in cases that it fails physicians can choose to either refer the patient to an interventional radiologist for sclerotherapy or perform a revision operation themselves. For this case, sclerotherapy was chosen and even after multiple rounds the TGDC and its symptoms persisted. Ultimately, a revision excision procedure was performed and the residual tissue was removed leading to the cessation of symptoms. Overall from the time of initial presentation until the final resolution of the patient’s symptoms, the treatment process spanned the course of 19 months. This case demonstrates the importance of a comprehensive evaluation and a careful consideration of alternative treatments when addressing the case of a recurrent TGDC. This is especially true when considering the extended treatment timeline and protracted duration of symptoms that are observed in this case.
